# Spatial and temporal dynamics of malaria transmission in rural Western Kenya

**DOI:** 10.1186/1756-3305-5-86

**Published:** 2012-04-28

**Authors:** Nyaguara Amek, Nabie Bayoh, Mary Hamel, Kim A Lindblade, John E Gimnig, Frank Odhiambo, Kayla F Laserson, Laurence Slutsker, Thomas Smith, Penelope Vounatsou

**Affiliations:** 1 Kenya Medical Research Institute/Centers for Disease Control and Prevention (CDC) Research and Public Health Collaboration, P.O. Box 1578, Kisumu, Kenya; 2 Swiss Tropical and Public Health Institute, Socinstr. 57, P.O. Box, 4002, Basel, Switzerland; 3 University of Basel, Petersplatz 1, P.O. Box 4003, Basel, Switzerland; 4 Centers for Disease Control and Prevention, 1600 Clifton Rd,. Atlanta, GA 30301, Georgia, USA; 5 Department of Epidemiology and Public Health (EPH) Swiss Tropical and Public Health Institute, Basel, Switzerland

## Abstract

**Background:**

Understanding the relationship between *Plasmodium falciparum* malaria transmission and health outcomes requires accurate estimates of exposure to infectious mosquitoes. However, measures of exposure such as mosquito density and entomological inoculation rate (EIR) are generally aggregated over large areas and time periods, biasing the outcome-exposure relationship. There are few studies examining the extent and drivers of local variation in malaria exposure in endemic areas.

**Methods:**

We describe the spatio-temporal dynamics of malaria transmission intensity measured by mosquito density and EIR in the KEMRI/CDC health and demographic surveillance system using entomological data collected during 2002–2004. Geostatistical zero inflated binomial and negative binomial models were applied to obtain location specific (house) estimates of sporozoite rates and mosquito densities respectively. Model-based predictions were multiplied to estimate the spatial pattern of annual entomological inoculation rate, a measure of the number of infective bites a person receive per unit of time. The models included environmental and climatic predictors extracted from satellite data, harmonic seasonal trends and parameters describing space-time correlation.

**Results:**

*Anopheles gambiae* s.l was the main vector species accounting for 86 % (n = 2309) of the total mosquitoes collected with the remainder being *Anopheles funestus*. Sixty eight percent (757/1110) of the surveyed houses had no mosquitoes. Distance to water bodies, vegetation and day temperature were strongly associated with mosquito density. Overall annual point estimates of EIR were 6.7, 9.3 and 9.6 infectious bites per annum for 2002, 2003 and 2004 respectively. Monthly mosquito density and EIR varied over the study period peaking in May during the wet season each year. The predicted and observed densities of mosquitoes and EIR showed a strong seasonal and spatial pattern over the study area.

**Conclusions:**

Spatio-temporal maps of malaria transmission intensity obtained in this study are not only useful in understanding variability in malaria epidemiology over small areas but also provide a high resolution exposure surface that can be used to analyse the impact of transmission on malaria related and all-cause morbidity and mortality.

## Background

Malaria parasites are transmitted from human to human via the bite of an infected female anopheline mosquito. The life cycle of the mosquito vector and the malaria parasite are strongly influenced by climatic factors, primarily rainfall, temperature and humidity. Suitable rainfall provides mosquito breeding sites and temperature influences both vector and parasite development. By understanding the relations between environmental/climatic factors and malaria transmission in space and time, transmission intensity can be estimated in areas where data are otherwise lacking and high risk areas can be identified. Understanding spatial and temporal variation in vector density and transmission intensity is useful in planning effective malaria control programs and determining the optimal allocation of limited resources.

Malaria transmission intensity is often assessed by the entomological inoculation rate (EIR) which is the product of the vector biting rate and the sporozoite rate (SR) which is the proportion of mosquitoes with sporozoites in their salivary glands [1]. EIR estimates the number of infective bites a person receives per unit time and thus the level of exposure of an individual to malaria parasites. Studies have shown strong correlation between EIR and malaria prevalence [2-4]. Furthermore, EIR is the most accurate measure of transmission intensity [5] particularly when efforts are made towards reducing human-vector contact.

Mosquito population size and sporozoite rates fluctuate between seasons and over years [6]. Shortly after the onset of rainfall, mosquito populations increase to a peak. As the dry season sets in, mosquito populations decline in numbers since no new recruits are added to the population [7]. A change in either mosquito density or sporozoite rate or both affects the EIR. Similarly, mosquito population distribution is heterogeneous [8-10] and even within a defined geographical area mosquito densities vary widely in space and time.

In the KEMRI/CDC Health and demographic surveillance systems (HDSS), entomological data are collected from randomly selected locations (houses) as part of routine surveillance to assess the effects of interventions aimed at reducing malaria transmission intensity. The main characteristics of the data are the presence of spatio-temporal correlation and the large number of locations without mosquitoes (zeros). Spatial correlation arises because neighbouring locations are influenced by similar exposures such as climate and environment due to close proximity of locations. Analyzing these data without taking into account these specific characteristics result in overestimation or underestimation of the statistical significance of the covariates [11] and poor model fit respectively.

Several studies have reported large spatio-temporal variations in mosquito density, SR and EIR [8,10,12,13]. For instance Dery et al. [12] reported sporozoite rate of 1.5 % and 4.7 % for *An. funestus* and *An.gambiae* respectively and annual EIR estimates of 267 and 231 infectious bites per person per year (ibpy) for first year and second year respectively in a study in the forest-savannah transitional belt of Ghana. Drakeley et al. [8] also reported SRs of less than 1 % with EIRs ranging from 4 to 108 in the cool and wet seasons respectively in Ifakara, a semi-urban area in Tanzania. In the same study, an EIR of 54 ibpy was reported in the eastern part of Ifakara town compared to only 15 ibpy at the center of the town. Smith et al. [10] mapped mosquito (*An. funestus* and *An.gambiae*) densities in Namawala, a single village in Morogoro region of south eastern of Tanzania. Overall, the spatial pattern of mean log densities of both species was similar with higher density of *An. funestus* in the southern edge of the village adjacent to rice growing fields. In the above studies, a large number of locations had zero mosquitoes. However, appropriate statistical methods taking into account zero inflation were not used to assess variation in space and time. In addition, sporozoite rate are binomial data, whereas mosquito densities are count data which requires different modeling approaches to obtain EIR.

In our previous work [14], we developed spatio-temporal zero inflated models to analyze sparse sporozoite rate data. These models have been used to obtain spatially explicit estimates and maps of sporozoite rates in the KEMRI/CDC HDSS. In this study we extend our previous work by analyzing zero inflated mosquito density data. Spatio-temporal model based-estimates of mosquito density are combined with sporozoite rate model based-estimates obtained by [14] to estimate the space-time pattern of EIRs.

## Methods

### Study site

**Figure 1:**
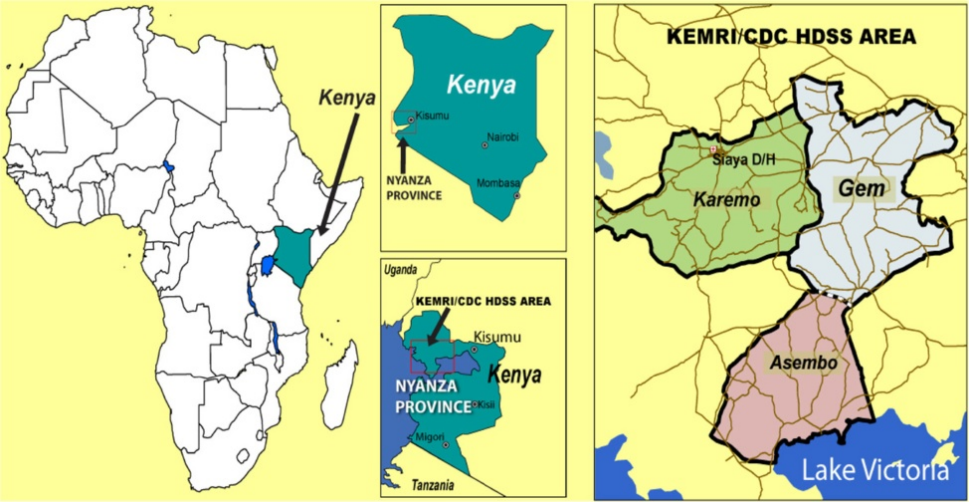
Location of the KEMRI/CDC HDSS site.

This study was carried out in the KEMRI/CDC HDSS site located in Asembo (Rarieda Division, Bondo District), Gem (Yala and Wagai Divisions, Siaya District) and Karemo (Karemo Division, Siaya District) areas situated in Nyanza Province, rural Western Kenya (Figure 1).

During the study period, the KEMRI/CDC HDSS was only operating in Asembo, bordering Lake Victoria and Gem, adjacent to and North of Asembo. The HDSS has been described elsewhere in detail [15]. In brief, KEMRI/CDC HDSS area is characterized by gentle hills/slopes (elevation =1,147-1,388meters) that are drained by several small streams and one permanent river in Gem. Rainfall occurs year-round with heavy rains falling from March through May and from November to December (wet season). The remaining months of the year receive only light showers (dry season). Most inhabitants reside in traditional houses with mud walls and thatched roofs clustered into compounds. The compounds consist of clusters of one or more houses separated from other such clusters by the surrounding agricultural fields. At the time the data were collected, the study area covered approximately 500 km^2^ with a population of 135,000 living in 33,990 households within 21,477 compounds.

Malaria is holoendemic in the KEMRI/CDC HDSS area where it is transmitted by *An.gambiae* s.l. and *An. funestus*[15,16]. A trial of insecticide-treated mosquito nets (ITNs) trial conducted from 1996 to 2002 reduced malaria transmission by 90 % [17,18]. However, despite the continued use of ITNs and a relatively low EIR of about seven ibpy [15], malaria prevalence remains high and is still the main cause of child mortality [15].

### Entomological data

The entomological data (2002–2004) used in this study has been described elsewhere in detail [14,15]. In brief, *Anopheles* mosquitoes were collected monthly using Centers for Disease Control (CDC) light traps from 10 randomly selected houses (locations) each month from HDSS database along with four additional houses neighboring each index house. In each house, a light trap was placed next to the sleeping place of an individual who was randomly chosen from the list of household members and mosquitoes were collected for two sequential nights. The sleeping place of the selected individual was covered with an insecticide treated net to protect the person from mosquito bites. Captured mosquitoes were initially identified morphologically while members of *Anopheles gambiae* complex were further identified to species using polymerase chain reaction (PCR) [19]. Female *Anopheles* mosquitoes were tested for the presence of circumsporozoite antigens using an enzyme linked immunosorbent assay method [20].

### Entomological inoculation rate (EIR)

The entomological inoculation rate (EIR) was calculated as the product of light trap densities and the proportion of infected mosquitoes (sporozoite rate). Mosquito density in the light traps was calculated by dividing the number of mosquitoes caught by the CDC light traps by the number of trap-nights. This estimate was then adjusted by multiplying by 1.605 as described by Lines and colleagues to calibrate the light trap estimates to those of human landing catch [21]. The conversion factor adjusts for vector collection bias between human bait catch technique which is directly associated with mosquito feeding on humans and light trap collection which tends to underestimate the densities observed in human landing catches [22]. High EIR resolution was obtained as a product of predicted mosquito SR and density at locations where mosquitoes were not collected. The former was extracted from analysis in [14].

### Climatic and Environmental data

The climatic and environmental predictors used in this study are similar to the ones used by Amek et al. [14]. Land surface temperature, normalized difference vegetation index, rainfall, and elevation were extracted from remote sensing data. Distance to the nearest water source (the lake, streams and river) was obtained from the KEMRI/CDC HDSS global positioning system (GPS) database.

Land surface temperature for day and night (LST) and Normalized Difference Vegetation Index (NDVI) were extracted at 0.25 km by 0.25 km and 1 km by 1 km spatial resolution respectively from Moderate Resolution Imaging Spectroradiometer (MODIS). NDVI is a proxy measure of vegetation cover ranging from 1 to −1. Positive values indicate the presence of vegetation and negative values and values close to zero represent barren soil or water surfaces.

Elevation (distance above the sea level) data were extracted at 1 km resolution from a Digital Elevation Model (DEM). MODIS and DEM were obtained from U.S Geological Survey (USGS) EROS Data Center. Rainfall estimate (RFE) data with an 8 km by 8 km spatial resolution from Meteosat 7 satellite were also obtained from the Africa Data Dissemination Service (ADDS).

All environmental factors were extracted for each location and lags up to 3 months were created to account for possible elapsing (lag) time, between the predictive variables (rainfall, LST and NDVI) and the outcome variable (mosquito density).

### Statistical analysis

The lag time analysis was carried out in STATA (version 9.0) to determine the best combination of lags that estimated the mosquito population density taking into account seasonality, distance to water bodies and elevation. Seasonality was modeled by (i) trigonometric functions with a cycle of 12 months [23] corresponding to two transmission seasons (wet vs. dry) and (ii) a binary variable indicating wet or dry season. The wet and dry seasons were defined based on rainfall data, with the months of March through May and November to December classified as the wet season, and the remaining months classified as the dry season. Trigonometric functions estimate the magnitude and the exact peak point (e.g. month, week or day) of the seasonal variation using the amplitude and the phase parameters respectively.

The Akaike’s information criterion [24] was used to select the best fitting model combining seasonality and environmental factors. The best model included seasonality with a cycle of 12 months, average NDVI and night temperature (LSTN) during the month of mosquito collection, average day temperature (LSTD) during the current and previous month of mosquito collection and total rainfall during the current and two previous months of mosquito collection. A Bayesian geostatistical version of the above model using a zero-inflation formulation was further fitted to assess space time variation. The model included year effect and an autoregressive term to take into account temporal correlation. Bayesian Kriging, similar to that used in our previous work [14] was used to predict mosquito density at locations (houses) where mosquitoes were not collected. Location specific predictions of sporozoite rate obtained by [14] and density were multiplied to obtain the EIR estimates

The assessment of model predictive ability was also similar to that carried out by [14]. We assessed model predictive ability by fitting the models on a training set of 85 % (943) of the randomly selected locations and compared the model-based predictions with the observed data at the remaining 15 % (167) test locations [25]. The best model was one with the highest percentage of test locations falling within the Bayesian credible interval of smallest coverage as well as the model with the smallest mean square error.

The Bayesian model was fitted in OpenBUGS version 3.1.2 (Imperial College and Medical Research Council, London, UK) and Kriging was carried out in a code written by the authors in Fortan 95 (Digital Equipment Corporation) using standard numerical libraries (Numerical Algorithms Group Ltd). A description of the Bayesian geostatistical formulation model fitted to mosquito count data is given in the appendix.

## Results

### Abundance/density of vector species

**Figure 2:**
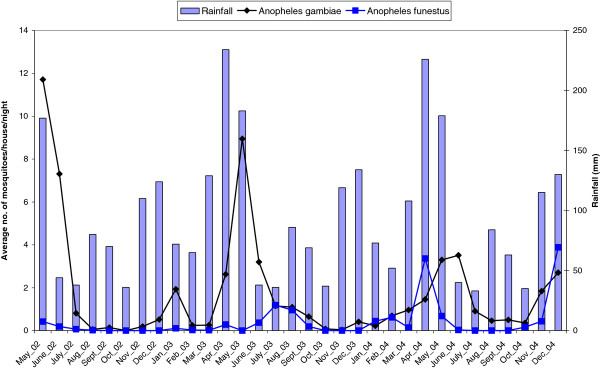
Monthly pattern of average number of Anopheles gambiae and funestus species in relation to total Rainfall.

A total of 2309 anopheline mosquitoes were collected from 3850 catches in 1110 unique locations during the study period. About 68 % of these locations had no mosquitoes. *An. gambiae* s.l. mosquito was the predominant vector species accounting for 86 % of the total *Anopheles* mosquitoes collected. The remaining 14 % were *An. funestus.* Average monthly abundance of *Anopheles* mosquitoes varied over the study period. Each year, the peak collecting period for *An. gambiae* was May, during the rainy season (Figure 2). *An. funestus* was very low throughout the study period except in the months of April and December in the year 2004. PCR tests on the *An. gambiae* s.l. samples indicated that the majority (72 %) were *An. gambiae* s.s with *An. arabiensis* accounting for the rest of the tested mosquitoes.

**Figure 3:**
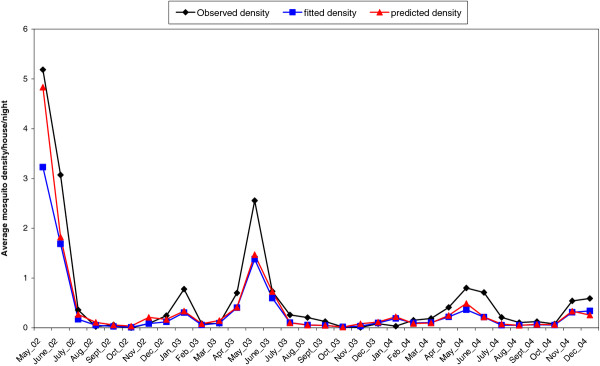
Monthly pattern of observed, fitted and predicted density of Anopheles gambiae mosquito.

Figure 3 shows the monthly pattern of observed, fitted and location-specific predicted density of *An. gambiae*. It should be noted that the observed density has a similar pattern to the location-specific predicted and fitted densities throughout the study period. *An. gambiae* density varied over the months with peaks in May of each year. However, the absolute density during the peak month (May) significantly decreased over the 3 years of the study. Comparison between wet and dry months indicated that density was higher in wet months.

**Figure 4:**
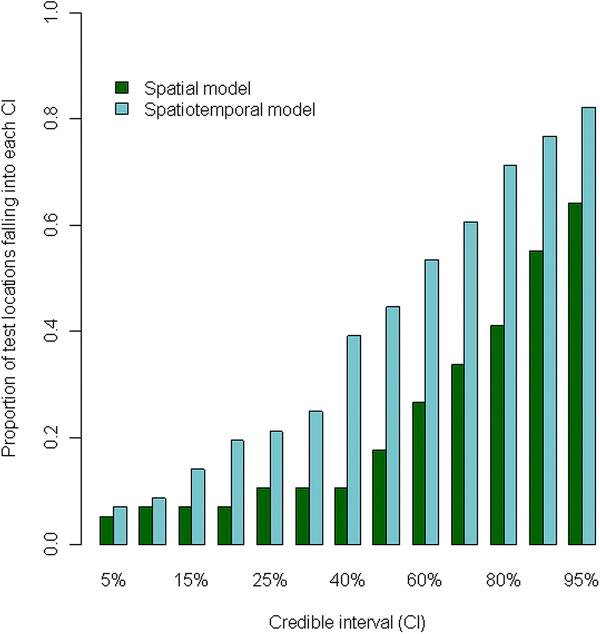
Proportion of test locations with none-zero mosquitoes falling in between 5 % to 95 % credible intervals of the posterior predictive distribution.

Model validation showed that 83 % and 66 % of the test locations had mosquito densities which were within the 95 % credible intervals estimated from the zero inflated spatio-temporal negative binomial model and zero inflated spatial negative binomial model respectively. Furthermore, the zero inflated spatio-temporal negative binomial model consistently included the highest proportion of test locations in all the credible intervals compared to spatial negative binomial model (Figure 4). Similar results were obtained using the mean square error measure (data not shown).

The best fitting zero-inflated spatiotemporal model included the following parameters: distance to water bodies, elevation, average value of NDVI and LSTN during the month of mosquito collection, average LSTD during the current and the previous month of mosquito collection, total rainfall during the current and the two previous months of mosquito collection, year trend, trigonometric seasonality, spatial and temporal variations. The results of bivariate non-spatial and spatio-temporal zero-inflated negative binomial models are shown in Table 1 below.

**Table 1:** Posterior estimates of zero inflated geostatistical density models

Covariates	Bivariate non-spatial	Spatiotemporal model
	Mean (95 % CI)	Median (95 % CI)
Intercept	-	4.634 (0.005,7.098)
Distance to water body	−0.003 (−0.006,0.001)	−0.007 (−0.013,-0.002)
Elevation	0.002 (−0.001,0.003)	−0.008 (−0.041,0.020)
Rainfall ^***^	0.006 (0.005,0.008)	0.040 (−0.041,0.113)
NDVI^*^	4.837 (3.589,6.086)	4.170 (1.308,6.725)
LSTD**	−0.139 (−0.182,-0.096)	−0.246 (−0.3752,-0.153)
LSTN^*^	−0.010 (−0.065,0.085)	0.124 (−0.031,0.234)
Year2	−0.276 (−0.538,-0.013)	0.242 (−0.356,0.852)
Year3	−0.404 (−0.673,-0.135)	0.441 (−0.244,1.122)
Cosine	0.642 (0.477,0.807)	1.75 (0.570,2.913)
Sine	0.533 (0.364,0.701)	0.522 (−0.597,1.590)
Amplitude	-	1.922 (0.941,3.016)
Shift/phase	-	0.280 (−0.291,1.033)
Over dispersion value	-	0.705 (0.502,1.135)
Spatial Variation	-	0.874 (0.516,1.417)
Temporal variation	-	0.322 (0.140,0.898)
Range(3/(*ρ*)^a^	-	3.039 (1.337,6.482)
Zero-Inflated proportion	-	0.074 (0.004,0.200)

a: minimum distance in kilometers at which spatial correlation is significant at 5 %,*: environmental average value of the current month of mosquito collection, **: environmental average value of the current and previous month of mosquito collection, ***: environmental total value of the current and two previous month of mosquito collection.

Distance to water bodies, mean value of NDVI during the month of collection and average day temperature during the current and the previous month of collection were associated with mosquito density. In particular, distance to water bodies and average day temperature (LSTD) during the current and the previous month of mosquito collection were negatively related with mosquito density. Mean value of NDVI during the month of collection was positively associated with mosquito density. The average of the total rainfall during the current and the two previous months of mosquito collection, mean night temperature (LSTN) during the month of collection and elevation were not associated with mosquito density. The minimum distance at which the spatial correlation was significant at 5 % was 3.0 km (95 % credible interval: 1.337, 6.482).

The 95 % credible interval of the amplitude parameter revealed a strong monthly variation in mosquito density. The phase of 0.28 radials indicated that the maximum density occured in the months of May and the minimum in November. However, the average mosquito density during the second and third year was not strongly different than that of the first year.

### Entomological inoculation rate

The overall point estimates of annual EIR were 6.7, 9.3 and 9.6 ibpy for the years 2002, 2003 and 2004 respectively. The estimates of EIR for this study were obtained exclusively from the *An. gambiae* mosquitoes because none of the *An. funestus* mosquitoes tested positive for the presence of *Plasmodium falciparum* sporozoite antigens. The estimates of EIR for 2002 are based on data from Asembo only since mosquito collection started in Gem in 2003. Gem had high EIR in both wet and dry seasons throughout the study period (Table 2 below).

**Table 2:** Distribution of EIR by area in relation to wet and dry months during study period

Area	2002		2003		2004	
	Wet	dry	Wet	Dry	Wet	dry
Asembo	4.9	1.8	4.3	2.8	4.9	1.2
Gem	-	-	6.6	4.9	8.5	4.6

**Figure 5:**
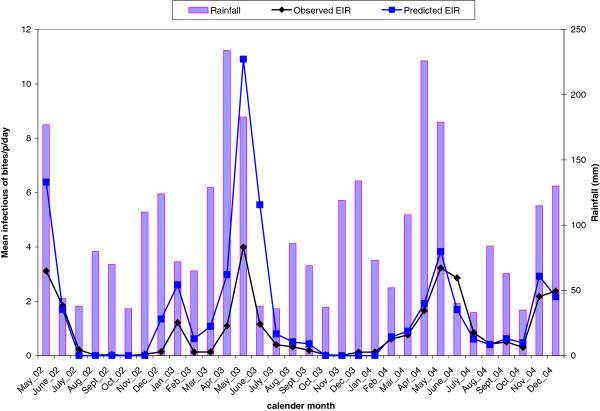
Temporal pattern of observed and predicted entomological inoculation rate in relation to rainfall during the study period.

Figure 5 depicts the temporal pattern of observed and predicted EIR in relation to total monthly rainfall. Overall observed and predicted EIR display a similar trend during the study period. However, our model over-predicted EIR in May 2002, May 2003 and June 2003. Monthly point estimates of EIR varied over the study period with the highest inoculation rate of 4 ibpm (infectious bites per person month) occurring in May 2004 and the lowest in October 2002. Comparison between wet and dry months indicated that both the predicted and observed EIR are higher during the wet months (data not shown).

**Figure 6:**
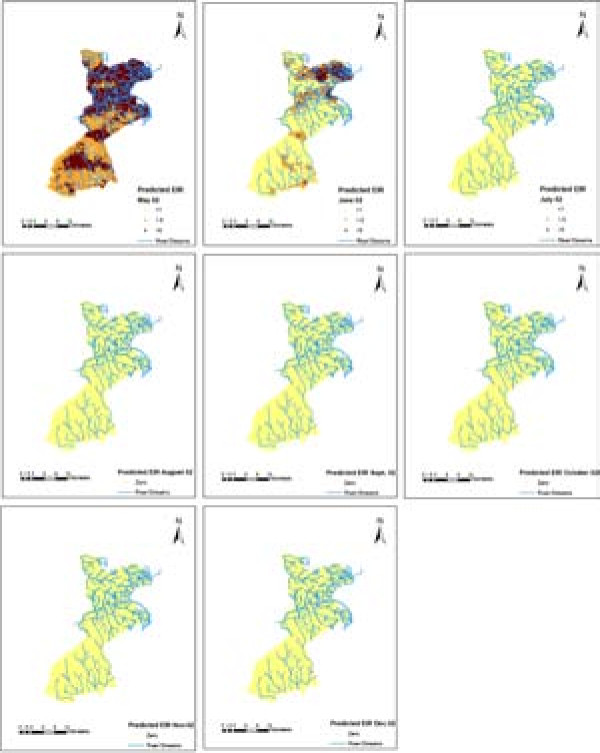
Predicted EIR maps for 2002.

**Figure 7:**
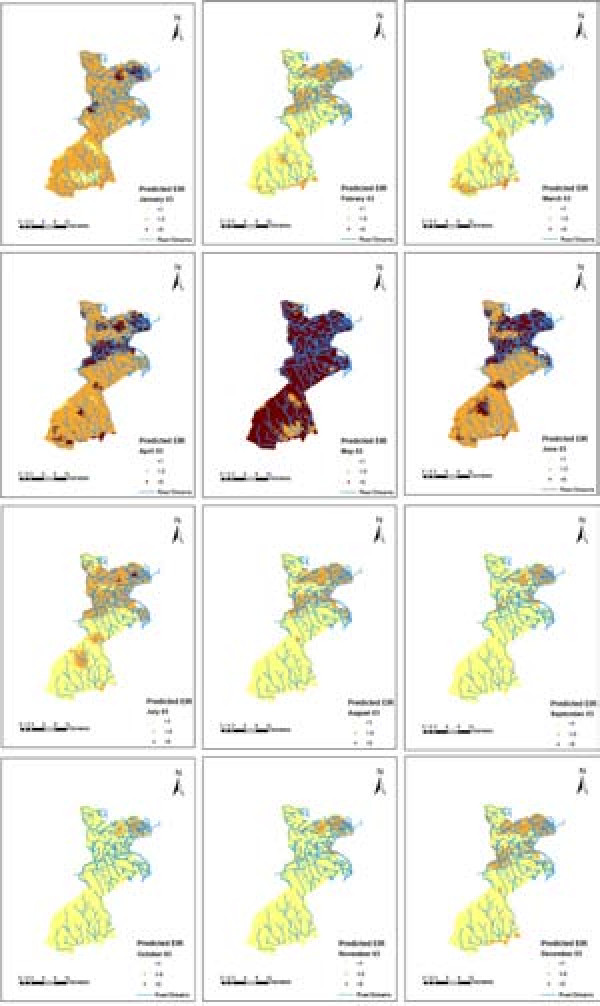
Predicted EIR maps for 2003.

**Figure 8:**
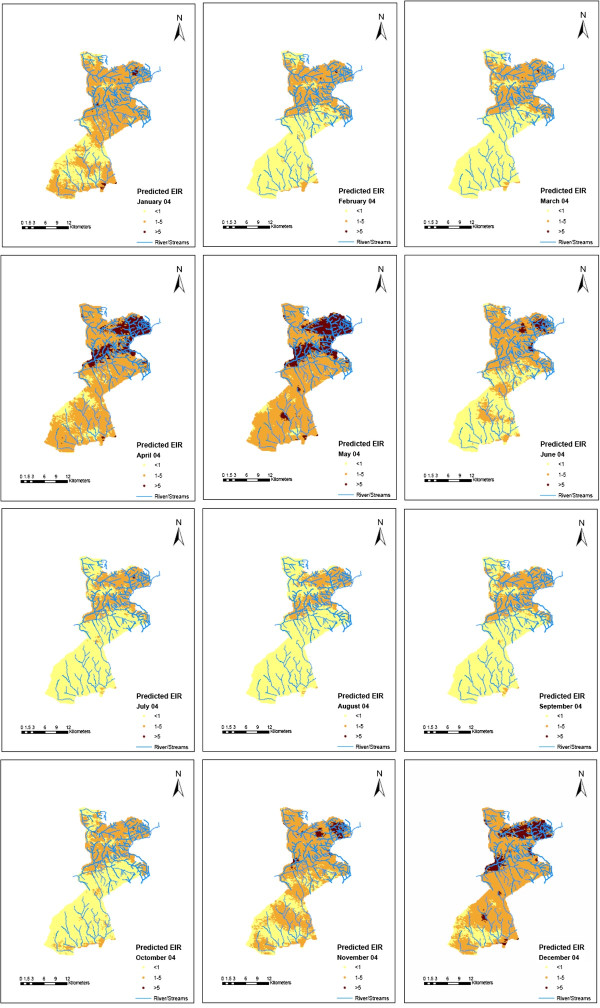
Predicted EIR maps for 2004.

Smooth maps of monthly predicted malaria transmission are shown in Figures 6, 7 and 8. These predicted maps depict spatial variation within and between months with areas of high predicted EIRs occurring in the northern part of the study area with a few locations with high predicted EIRs occurring during wet months in the southern part of the study area. Prediction error maps (not shown) were also produced.

## Discussion

In this study, we described and estimated malaria transmission patterns in the KEMRI/CDC HDSS site using mosquito density and entomological inoculation rate as measures of malaria transmission intensity. Malaria transmission fluctuated over the months (see Figure 2) in the HDSS with the highest mosquito density/abundance and EIR occurring in May each year. Transmission intensity measured by EIR showed that residents in the HDSS were exposed to a range of 0–4 infective bites per month (see Figure 4). The results also showed that *An. gambiae* is the main species driving transmission because the density and infectivity of *An. funestus* were very low. *An. gambiae* prefers temporary breeding sites which are common in the study area during the rainy seasons as opposed to *An. funestus* which is mainly found in permanent water bodies. A study [26] on the impact of ITNs on entomological indices in the same area also found a similar result (high density of *An. gambiae* compared to *An. funestus*).

The negative association between distance to water bodies and mosquito density in our results implies that many mosquitoes tend to be found close to the water bodies that act as the breeding sites. This probably applies to both newly emerged mosquitoes and adult mosquitoes that have limited dispersal ability. A study of the geographic distribution of adult mosquitoes in the same area also found a significant relationship with water bodies identified in a GIS database during the dry season [9]. Although elevation was negatively associated with mosquito density, the relationship was not strong.

Temperature is an important factor related to mosquito development and survival and to the duration of the sporogonic cycle of the parasite [27]. Temperatures above 22^o^ C are suitable for stable malaria transmission [28] and in our study area, the average daily maximum temperature is about 29^o^ C. In our study, the average day temperature during the current and the previous month of mosquito collection had a strong negative effect on mosquito density.

NDVI, a proxy measure of vegetation was positively associated with mosquito density. The higher the NDVI value the greener the vegetation which is suitable for mosquito development.

The spatial correlation in mosquito density was strong at distances up to about 4 km (95 % credible interval: 2.044, 11.370). However, a study by Midega and colleagues found a maximum distance of mosquito dispersal of only 0.7 km using a capture-recapture technique at the Kenyan coastal region [29]. Mosquito dispersal is unlikely to explain this rather long distance correlation, which is probably due to unobserved/unmeasured spatially-correlated factors such as the spatial pattern of breeding sites and possibly socioeconomic status.

The smooth maps generated in this study show that malaria transmission intensity in the HDSS varies over space and time, with high transmission occurring in a few pockets (hot spots). EIR peaks shortly after the onset of the long rains in May of each year. Comparison between the study regions shows that EIR is consistently higher in Gem than Asembo which may be attributable to the occurrence of more rivers and streams in Gem that contribute to the creation of large numbers of mosquito breeding sites. Similarly, substantial differences in the overall EIR between 2002 and 2003 could be due to earlier interventions in some parts of Asembo [26].

Most analyses of mosquito sporozoite rate, density and EIR in relation to environmental/climatic factors and/or malaria incidence have been based on the assumption of independence between observations. However, mosquito data are usually collected repeatedly over time at fixed geographical locations thus are spatially correlated due to common exposures. Similarly, mosquito density data are count data which are commonly analysed using the Poisson distribution. However, the Poisson distribution assumes that the mosquito average equals the variance which is not always the case with entomological data which usually has a large number of locations with zero mosquitoes even in areas of high transmission. Our proposed Bayesian geostatistical zero-inflation model for assessing the relationship between mosquito density and environmental/climatic factors takes into account the underlying spatial processes and overdispersion associated with observed “excess zeros”. The model has a large number of parameters. However, simulation-based Bayesian computation allows simultaneous estimation of all parameters including the error of the location-specific predictions, a feature missing in the maximum likelihood based framework.

Our work used variable selection method based on standard models. Geostatistical variable selection has been applied in malaria epidemiology [30]. However, this method could not be employed in our data which was collected over large number of locations. We are currently developing methodology to address this problem.

## Conclusions

The maps of EIR produced in this study provide a high resolution exposure surface which is useful in analyzing the impact of transmission on malaria related and all-cause morbidity and mortality. At the same time, these maps help us understand the variability in malaria epidemiology over small areas.

## Competing interests

The authors declare that they have no competing interests.

## Authors’ contributions

NA conceptualized the statistical method, analyses and interpretation of data, and drafted the paper. NB, KAL and JEG conceived and designed of the entomological study and revised the draft. MH, FO, KFL, LS, and TS helped in interpretation of data and critically revised the draft for intellectual content. PV conceptualized the statistical modeling, interpreted the data and critically revised the drafts for intellectual content. All authors read and approved the final manuscript.

## Appendix A

### Negative binomial and Zero-Inflated negative binomial models

Mosquito density data are typical overdispersed count data, thus modeled using the negative binomial model: Let *Y*_* it *_ be the number of mosquitoes at location *ί* at time * t *, arising from a negative binomial distribution; $Y_{i}{}_{t}\sim NB(\mu_{i}{}_{t},r)$ with mean $\mu_{i}{}_{t}$ and parameter *r* measuring the extra variation (overdispersion) in our data. To capture the excess zero values that cannot be accounted for by the overdispersion parameter *r*, we used the zero-inflated negative binomial (ZINB) model which is a mixture model with two components: one ar∈g from the negative binomial distribution and the other corresponds to the excess zeros. That is

$f(Y_{i}{}_{t}=y_{i}{}_{t})\sim \{\begin{array}{l} 0\;with\;probability\;p_{i}{}_{t}\\ NB(\mu_{i}{}_{t}\text{,}\;r)with\;probability\;1-p_{i}{}_{t}\; \end{array}$.The ZINB density is given by $f(y_{i}{}_{t})=(1-p_{i}{}_{t})\frac{(y_{i}{}_{t}+r-1)!}{y_{i}{}_{t}!(r-1)!}{\left(\frac{r}{r+\mu_{i}{}_{t}}\right)}^{r}{\left(\frac{\mu_{i}{}_{t}}{r+\mu_{i}{}_{t}}\right)}^{y_{i}{}_{t}}\text{,}\;r>0\;\text{,}\;and\;y_{i}{}_{t}>0$ with the mean equal to $(1-p_{i}{}_{t})\mu_{i}{}_{t}$ and the variance given by var$(Y_{i}{}_{t})=(1-p_{i}{}_{t})\left(1+\frac{\mu_{i}{}_{t}}{r}+p_{i}{}_{t}\mu_{i}{}_{t}\right)\mu_{i}{}_{t}$. The term $p_{i}{}_{t}$ is the mixing proportion. The above model reduces to zero inflated Poisson distribution as r→∞. The environmental and seasonality factors, ${\underline{X}}_{i}{}_{t}$ were modeled on the log$\left(\mu_{i}{}_{t}\right)$ scale of the mean of the outcome, that is log$\left(\mu_{i}{}_{t}\right)$=${\underline{X}}_{i}{{}_{t}}^{T}\underline{\beta }$, where $\underline{\beta }$ is the vector of regression coefficients.

### Geostatistical zero inflated negative binomial model

Mosquito data used in our analysis are collected at fixed geographical locations, sharing common exposures such as environmental and climatic factors thus correlated in space. To take into account the spatial correlation, we introduce spatial correlation parameter by adding location-specific random effect ${\mathrm{phi}}_{i}$ on the mean structure of the above model: log$\left(\mu_{i}{}_{t}\right)$=${\underline{X}}_{i}{{}_{t}}^{T}\underline{\beta }+{\mathrm{phi}}_{i}$. We further assume that random effects are parameters from a latent Gaussian spatial process with covariance matrix and model spatial correlation between any pair of locations as a function of their distance irrespective of the direction. We used an exponential correlation function, that is$\underline{\Phi }={\left(\Phi_{1,}........,\Phi_{n}\right)}^{T}\sim N(0,\Sigma )$$\Sigma_{\mathrm{ij}}=\Sigma_{1}{}_{}^{2}$ corr$\left(d_{\mathrm{ij}},Ρ\right)$ where $\Sigma_{1}{}_{}^{2}$ is the spatial variation, $d_{\mathrm{ij}}$ is the distance between location * ί * and *j*, and * ρ *is the smoothing parameter, measuring the rate of correlation decay with increasing distance. The value 3/Ρ estimates the minimum distance at which spatial correlation is less than 5 % [31].

In addition to the above spatial correlation, mosquitoes were collected monthly in different locations during the study period and thus correlated in time too. We model temporal correlation by introducing monthly random effects ($\in_{t}$) to the above model:$\log(\mu_{i}{}_{t})={\underline{X}}_{i}{{}_{t}}^{T}\underline{\beta }+{\mathrm{phi}}_{i}+\in_{i}{}_{t}$ and modeled by autoregressive (AR) process of various orders. The deviance information criterion (DIC) [32] was used to identify the best fitting order of the process, which was found to be one. Thus we considered that $\in_{t}\sim N(\gamma \in_{t-1},\Sigma_{2}^{2})$ and ∈1~N(0,Σ221−γ). The terms $\Sigma_{2}^{2}$and *γ* are the temporal variance and autocorrelation parameters respectively with γ∈(−1,1).

### Model fit

Prior distributions of the above model parameters were adopted to complete the Bayesian model specification above. In particular, we choose non-informative Normal prior distribution for the $\underline{\beta }$ parameters with large variance, an inverse gamma priors for $\Sigma_{1}{}^{2}$ and $\Sigma_{2}^{2}$, a gamma prior for * ρ * and a Uniform prior for *γ*, that is $\Sigma_{1}{}^{2},\Sigma_{2}^{2}\sim IG(2.01,1.01)$γ~U(−1,1) andΡ~G(0.1,0.1). We further consider a constant zero-inflated mixing proportion across the area and time with a Uniform prior distribution π~U(0,1). To estimate the model parameters, we employed Markov Chain Monte Carlo (MCMC) simulation algorithm [33] and starting with some initial values about the parameters, we ran two chains sampler discarding the first 5000 iterations. Convergence was assessed by Gelman-Rubin diagnostic [34]. Using Bayesian Kriging [35] method that is for each sample of the parameters from the posterior distribution, a random effect is simulated from the Gaussian spatial process conditional on the random effects estimated at the observed locations. This is added on the regression term relating the covariates at the new location with the regression coefficients estimated during the model fit. The resulting equation estimates the mosquito density on the logit scale at the new location as a sample from the posterior predictive distribution. A grid of 7726 pixels with 250 meters by 250meters spatial resolution covering the entire study area was used to predict density.

The Bayesian model was fitted in OpenBUGS version 3.1.2 (Imperial College and Medical Research Council, London, UK). Bayesian Kriging was carried out in a code written by the authors in Fortan 95 (Digital Equipment Corporation) using standard numerical libraries (Numerical Algorithms Group Ltd).
